# Epidemiology of Accidental Injuries at Home and Related Risk Factors for Mortality among Older Adults in South Korea: A Retrospective Cohort Study

**DOI:** 10.3390/medicina60040593

**Published:** 2024-04-03

**Authors:** Ok-Hee Cho, Jeongeun Yoon

**Affiliations:** 1 Department of Nursing, College of Nursing and Health, Kongju National University, Gongju 32588, Republic of Korea; ohcho@kongju.ac.kr; 2 Department of Nursing, Kunsan College of Nursing, Gunsan 54068, Republic of Korea

**Keywords:** elderly, home accidents, injuries, mortality

## Abstract

*Background and Objectives*: Accidental home injuries among older adults are increasing globally, but reporting is limited. This study aims to establish foundational data for program development and policies to prevent accidental injuries at home in older adults by using data on the occurrence of accidental injuries at home and analyzing the risk factors of mortality due to accidental injuries among adults aged 65 years and older. *Materials and Methods*: This retrospective study used data from the community-based Severe Trauma Survey in South Korea. This study identified general, injury-related, and treatment-related characteristics of older adults who were transported to the emergency department with accidental injuries at home. Single-variable and multiple logistic regression analyses were used to identify risk factors for mortality after injury. *Results*: The majority of older adults in this study who experienced accidental injuries at home were aged 75 to 84 (42.8%) and female (52.8%), with 1465 injured from falls and slips (68.0%). Risk factors for mortality included older age (≥85 years) (ORs 2.25, 95% CI 1.47–3.45), male sex (ORs 1.60, 95% CI 1.15–2.20), mechanism of injury (falls or slips vs. contact injury, ORs 6.76, 95% CI 3.39–13.47; airway obstruction vs. contact injury, ORs 13.96, 95% CI 6.35–30.71), higher severity (moderate vs. mild, ORs 2.56, 95% CI 1.45–4.54; severe vs. mild, ORs 12.24, 95% CI 6.48–23.12; very severe vs. mild, ORs 67.95, 95% CI 38.86–118.81), and receiving a blood transfusion (ORs 2.14, 95% CI 1.24–3.67). *Conclusions*: Based on these findings, the home and community environments where older adults live should be inspected and monitored, and in-home accidental injury prevention strategies should be developed tailored to the characteristics of older adults’ risk factors and their injury-related characteristics.

## 1. Introduction

The older adult population is growing rapidly with improvements in healthcare quality, healthcare access, and drug development. The percentage of the global population aged 65 and older was expected to reach 9.3% in 2020 and is expected to reach 15.9% in 2050 [1]. With this demographic shift, trauma among older adults is emerging as a major public health issue [2]. In a cohort study at level I trauma centers in Canada, 35.1% of hospitalized patients were 65 years or older, and in-hospital mortality was higher in older patients [3]. In the United Kingdom (UK), the second-highest proportion of severe trauma patients were aged 75 and over, and the primary mechanism that led to major trauma was falls from a height of less than 2 m [2]. The United States (US) National Trauma Database reported an increase in the proportion of the older adult trauma population from 18% in 2005 to 30% in 2015 [4]. South Korea is no exception, and as of 2021, approximately 80% of patients hospitalized for injuries were adults aged 65 or older. The study revealed that 71% of patients aged 75 or older experienced incidents related to falls or slips, with 67.8% of falls occurring at home and 63.5% of slips also taking place in a home setting [5]. The Korea Disease Control and Prevention Agency (KDCA) has noted that in foreign countries, falls and slips of older adults are managed as injuries that should be prevented; however, in South Korea, the ministries concerned lack coordination and strategy to improve preventive measures for these injuries. Identifying the causes of accidental home injuries and factors that increase the risk of death can help determine priorities for injury prevention policies.

Older adults are more vulnerable to injury due to age-related physical changes, weakened motor and sensory functions, and a significantly reduced ability to cope with unexpected situations [4]. In addition, older adults’ perception of injury, the complexity of their surroundings, and the physical environment also contribute to major and minor trauma [6]. Meanwhile, older adults spend more time at home, experience difficulties including a loss of income due to retirement, a minimized role in society, declining health, and psychological isolation, all of which contribute to trauma [7]. Older adults in Korea have been reported to experience a relatively high poverty rate, and low socioeconomic conditions have been associated with an increased risk of accidental injuries such as falls [8]. In studies conducted in the US [9], Canada [3], Australia [10], the UK [2], and other countries, the most common causes of trauma in older adults were motor vehicle accidents and low- and high-level falls, with approximately one-fifth of accidental injuries occurring at home. The Centers for Disease Control and Prevention report that accidental injury is one of leading causes of potentially preventable deaths, including 43% of accidental injury deaths [11]. According to the Korea Consumer Agency’s 2022 Trend Analysis of Hazard Information for Older Adults, the home was the most common location for hazards in the last four years, with slips, trips, and falls accounting for most accidents. The study also found that risks from food consumption increased by 44.4% year-on-year [12]. This evidence suggests that understanding the nature and risk factors of injuries at home is necessary to minimize loss, disability, or death due to trauma, as well as protect the safety of older adults.

Previous studies of accidental injuries at home have reported that more than one-third of older adults fall in any given year and that fall mortality rates have steadily increased over the past decade, with higher rates among men. Injury from fire and burns was the sixth leading cause of traumatic death for people aged 65 and older. In addition, adults aged 85 and older were about four times more likely to die in house fires than younger adults were. Between 1999 and 2013, the mortality rate from drug poisoning more than doubled in adults aged 60 and older, and the increase in drug overdose deaths was greater in older women than in older men [13]. A study from Malaysia reported that women aged 60 or older, those aged 70–74, and those living alone were more likely to experience injuries in their homes. Moreover, there was a higher likelihood of injuries occurring in the households of older adults living alone compared with those of adults who were married [14]. While accidental injury at home is preventable, management approaches vary depending on the characteristics of the older adult population and the type of incident.

This study aims to provide foundational data for program development and policies to prevent accidental injuries at home in older adults. It focuses on examining the occurrence of accidental injuries at home and analyzing the risk factors of mortality due to accidental injuries among adults aged 65 years and older.

## 2. Materials and Methods

### 2.1. Study Design

This study was a retrospective, cross-sectional correlational study that secondarily analyzed national injury statistics in South Korea.

### 2.2. Population and Procedures

This study was approved for exemption from review by the Institutional Review Board and analyzed records from the Korean Community-based Severe Trauma Survey (CSTS) (Statistics Korea approval number 117105) from the KDCA, which excludes personally identifiable variables such as medical center code numbers and patient identification numbers. The CSTS is organized by the KDCA and is collected to build a foundation for the evaluation of the emergency medical system by producing statistics by region and emergency medical center, including the occurrence and treatment outcomes of severe injuries and multiple injuries. The CSTS is a surveillance system that captures the entire process, from injury to transportation, treatments, and outcomes. The data registered in the national-level surveillance system are subject to quality control by professional investigators and external experts to check and correct errors, and only data that have been quality controlled are released. These include the data on the total number of severe traumas, non-traumatic severe injuries, and multiple casualties transported by 911 ambulances annually, collected by professional investigators after confirming the patients through a mandatory record examination. Since data are based on patients who were transported to a medical center by a 911 ambulance, the study did not include patients with severe trauma who drove themselves or used a private ambulance. The survey items are based on international trauma registries, including the National Trauma Data Bank in the US and The Trauma Audit & Research Network in the UK, while considering the practicalities of data collection in South Korea.

**Figure 1:**
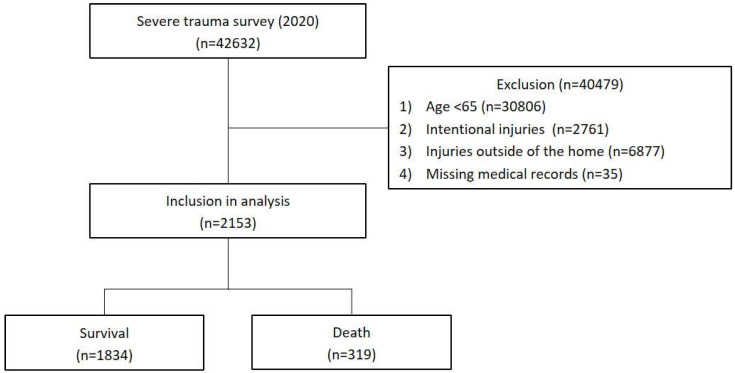
Flow chart of study population selection.

This study used data (released in June 2023) from patients injured during the period 1 January–31 December 2020. Data were collected from 15 emergency medical facilities (regional trauma centers, community emergency medical centers, etc.). Of the 42,632 records collected in 2020, the final analysis included a total of 2153 patients aged 65 and older with accidental injuries at home, excluding 30,806 under the age of 65, 2761 intentional injuries, 6877 injuries outside of the home, and 35 missing medical records (Figure 1).

### 2.3. Measurements and Scales

For this study, the following variables were obtained from the CSTS, investigated by the KDCA. This study identified the general, injury-related, and treatment-related characteristics of the subjects.

#### 2.3.1. General Characteristics

Sex

Age: The analysis was conducted by categorizing patients into three age groups: 65–74, 75–84, and ≥85 years old.

#### 2.3.2. Injury-Related Characteristics

Injury mechanism: Cases of injury resulting from contact with different substances or chemicals were classified as ‘contact injury’. Incidents involving tripping or slipping on the same surface, falling from a building, slipping on stairs, or falling down stairs were categorized as ‘fall or slip’. Instances of airway obstruction caused by inhaled objects or substances, as well as obstruction due to objects covering the mouth or nose, were classified as ‘airway obstruction’. Additionally, penetrating injuries, blunt injuries, thermal injuries, and unknown cause injuries were grouped under the category ‘others’.

Activity at the time of the incident: The activities of remote work, household chores, and cultivating a home garden or yard were classified as ‘working’, while activities related to relaxation, sleep, eating, drinking, washing, bathing, engaging in sexual activities, getting dressed, and other personal hygiene behaviors were categorized as ‘Daily living’. Wandering around the house or nearby areas without a specific purpose, walking, or running were classified as ‘on the move’, and activities such as exercising as a hobby, sitting, standing, and unknown activities were grouped under ‘others’.

Injury severity score (ISS): The ISS divides the body into six regions (head and neck, face, chest, abdomen, extremities, and body surface) and scores the severity of injuries in each region using the abbreviated injury scale (AIS). The scores are then summed by squaring the AIS scores of the three most severely affected regions. In cases where the score is unknown, it is classified as ‘unknown’. The scores range from 1 to 75, with 1–8 categorized as ‘mild’, 9–15 as ‘moderate’, 16–24 as ‘severe’, and 25 or more as ‘very severe’ [15].

#### 2.3.3. Treatment-Related Characteristics

Surgery: Including both emergency and non-emergency surgeries, if a surgical procedure was performed during the hospitalization period, it was classified as ‘Yes’, and if no surgery was performed, it was categorized as ‘No’.

Blood transfusion: Cases were classified as ‘Yes’ if we investigated only cases involving the transfusion of red blood cells, typically expressed in medical records as RBCs, packed cells, or packed red blood cells. The investigation covered transfusions conducted in various settings, including emergency rooms, intensive care units, operating rooms, and general hospital rooms. Other cases were classified as ‘No’.

### 2.4. Data Analysis

The collected data were analyzed using IBM SPSS Statistics 27.0 (IBM Corp., Armonk, NY, USA). Participant characteristics were identified using descriptive statistics such as actual numbers and percentages. Single-variable and multiple logistic regression analyses were used to identify risk factors for mortality according to the characteristics of patients with accidental injuries at home. A direct approach was utilized, inputting all independent variables into the model simultaneously without making assumptions regarding the order or relative value of variables. A *p*-value of 0.05 was considered statistically significant. Interactions were tested for the model, checking the Hosmer–Lemeshow test, classification table, and area under the curve. The model interpreted the ORs and 95% confidence intervals (95% CIs) to estimate the association between death and the independent predictors of mortality.

## 3. Results

### 3.1. Characteristics of Older Adults with Accidental Injuries at Home

In 2020, the accidental injury group included 2153 older adults, of whom 1834 survived and 319 died. Within the overall cohort, 697 (32.4%) were in the 65–74 age group, 921 (42.8%) were in the 75–84 age group, and 535 (24.8%) were aged 85 or older. In the mortality group, 96 (30.1%) were in the 65–74 age group, 133 (41.7%) were in the 75–84 age group, and 90 (28.2%) were aged 85 or older, highlighting that the highest mortality rates in both subjects and fatalities were within the 75–84 age group. Female older adults constituted 1137 (52.8%) of the total injured individuals, while male older adults accounted for 1015 (47.2%), indicating a higher proportion of female older adults. However, in the mortality group, there were 136 female older adults (42.6%) and 183 male older adults (57.4%), emphasizing a higher proportion of male older adults among fatalities.

The injury mechanism in the accidental injury group included falls and slips (*n* = 1465, 68.0%), followed by contact injuries (*n* = 317, 14.7%), other causes (*n* = 189, 8.8%), and airway obstructions (*n* = 182, 8.5%). In the mortality group, falls and slips were the most common (*n* = 168, 52.7%), followed by airway obstructions (*n* = 103, 32.3%), other causes (*n* = 30, 9.4%), and contact injuries (*n* = 18, 5.6%). Among all individuals, the majority of injuries occurred during ‘daily living’ (*n* = 1470, 68.3%), and this pattern was consistent in the mortality group (*n* = 203, 63.6%). Among the overall injured individuals, the injury severity scores (ISS) were distributed as follows: ‘mild’ injuries in 831 (38.6%), ‘moderate’ injuries in 547 (25.4%), ‘unknown’ in 383 (17.8%), ‘very severe’ injuries in 288 (13.4%), and ‘severe’ injuries in 104 (4.8%). In the mortality group, the distribution was as follows: ‘very severe’ injuries in 194 (60.8%), ‘unknown’ in 38 (11.9%), ‘moderate’ injuries in 36 (11.3%), ‘severe’ injuries in 29 (9.1%), and ‘mild’ injuries in 22 (6.9%). Overall, 371 individuals (17.2%) underwent surgery, and 145 (6.7%) received blood transfusions (Table 1).

### 3.2. Risk Factors for Mortality

Single-variable and multiple logistic regression analyses were utilized to identify the risk factors for mortality among older adults who experienced accidental injuries at home (Table 2). In the single-variable regression analysis, male older adults had 1.62 times higher odds of mortality compared with female older adults (95% CI = 1.27–2.06, *p* < 0.001). Injuries categorized as ‘fall or slip’ were associated with 2.15 times higher odds of mortality than ‘contact’ injuries (95% CI = 1.30–3.56, *p* = 0.003). Remarkably, ‘airway obstruction’ carried a staggering 21.66-fold higher likelihood of mortality than did ‘contact’ injuries (95% CI = 12.39–37.87, *p* < 0.001), while injuries categorized as ‘others’ had 3.13 times higher odds of mortality in comparison (95% CI = 1.69–5.80, *p* < 0.001). In terms of the ISS, the odds of mortality were 2.59 times higher for ‘moderate’ than for ‘mild’ severity scores (95% CI = 1.51–4.45, *p* = 0.001), and a substantial 14.22 times higher for ‘severe’ scores (95% CI = 7.78–25.97, *p* < 0.001). Furthermore, the ISS was 75.89 times higher for ‘very severe’ (95% CI = 46.50–123.87, *p* < 0.001) and 4.05 times higher for ‘unknown’ scores than for ‘mild’ scores (95% CI = 2.36–6.95, *p* < 0.001). Receiving a blood transfusion was associated with 2.83 times higher odds of mortality compared with not receiving one (95% CI = 1.94–4.13, *p* < 0.001), and 1.76 times higher odds for ‘unknown’ cases (95% CI = 1.04–2.98, *p* = 0.034). However, factors such as age, activity at injury, and surgery were not statistically significantly associated with mortality (Table 2).

In the multiple regression analysis, odds of mortality were 2.25 times higher in the 85 or older group compared with those in the 65–74 group (95% CI = 1.47–3.45, *p* < 0.001), and male older adults had 1.60 times higher odds of mortality compared with female older adults (95% CI = 1.15–2.20, *p* = 0.005). ‘Fall or slip’ injuries were associated with 6.76 times higher odds of mortality than ‘contact’ injuries (95% CI = 3.39–13.47, *p* < 0.001). ‘Airway obstruction’ had 13.96 times higher odds of mortality than ‘contact’ injuries (95% CI = 6.35–30.71, *p* < 0.001), and ‘others’ injuries had 4.18 times higher odds of mortality than ‘contact’ injuries (95% CI = 2.04–8.55, *p* < 0.001). In terms of the ISS, the odds of mortality were 2.56 times higher for ‘moderate’ than ‘mild’ injury severity scores (95% CI = 1.45–4.54, *p* = 0.001), and 12.24 times higher for ‘severe’ scores (95% CI = 6.48–23.12, *p* < 0.001). Furthermore, the odds of mortality were 67.95 times higher for ‘very severe’ scores (95% CI = 38.86–118.81, *p* < 0.001) and 14.45 times higher for ‘unknown’ scores than they were for ‘mild’ scores (95% CI = 7.38–28.27, *p* < 0.001). In addition, the odds of mortality were 2.14 times (95% CI = 1.24–3.67, *p* = 0.006) higher for those who received a transfusion compared with the odds for those who did not. Activity at injury and surgery were not statistically significantly associated with mortality (Table 2).

## 4. Discussion

As the older adult population expands, transitioning from a rapidly aging to a super-aged society [16], it is likely to be useful to understand the characteristics of the most frequent home injuries and factors associated with mortality. First, those aged 75–84 accounted for the majority of the accidental injury group, and the odds of mortality were the highest for those aged 85 or older. Second, while female older adults were more commonly injured, male older adults accounted for a larger proportion of mortalities. Third, most injuries were falls and slips, and airway obstruction led to higher odds of mortality than did other injuries. Fourth, higher ISS and transfusions were associated with higher odds of mortality.

The 75–84 group had the most accidental injuries and deaths in this study. However, the odds of mortality were higher for those aged 85 and older than for those aged 65 to 74. In previous studies, age was associated with increased post-traumatic mortality [17,18]. Older adults are at increased risk of mortality due to decreased physiological reserves, comorbidities, medications, and an increased risk of malnutrition [18]. In particular, around the age of 75, the rate of aging and psychological and physiological changes accelerate, leading to a decline in physical control [6]. Increased time spent at home contributes to the high incidence of injuries at home for older adults aged 75 to 84 [12]. In individuals over age 80, frailty is known to increase in-hospital mortality, even in the absence of significant trauma [19]. Frailty is a clinical condition characterized by a decline in homeostatic reserves and is associated with increased vulnerability to endogenous or exogenous stressors [20], increasing the risk of mortality from impairment [19]. A 16-year retrospective study of in 27,049 trauma patients aged 60 years and older reported that the number of trauma patients aged 80 years and older is increasing and that trauma patterns are changing [21]. Therefore, to prevent mortality, it is important to understand the age-specific lifestyles and behaviors of older adults.

In this study, women had a higher rate of injury at home, but more male older adults died as a result of injury, and the odds of mortality were higher for male than for female older adults. A previous study of 79,386 older adults in the US found that men had a higher mortality rate from accidental falls, both in and out of their homes, in line with the findings of this study [22]. It is interesting to note that older women account for more of the accidental injury group, whereas the risk of mortality is higher for older men, even though the proportion of women increases with age. Older men tend to have more chronic diseases and engage in more risky behaviors (smoking, drinking, substance abuse, and suiciding behaviors) than women of the same age [22]. It is also possible that older men are exposed to higher-risk situations compared with women, as they tend to engage in more risky activities or labor-intensive chores around their homes, including home repairs, heavy lifting, and using ladders [23]. Therefore, further data collection and analysis of the subjects’ medical history, medications, and levels of health risk behaviors are needed to identify specific sex differences related to injuries at home in older adults.

In this study, falls and slips accounted for the largest proportion of injuries, with odds of mortality about six times higher than those for contact injuries from chemicals. Falls are known to be one of the leading causes of hospitalization and mortality in older adults [24,25], and the results of this study support this. According to the Korea Consumer Agency, Fair Trade Commission & Rural Development Administration in 2022, falls accounted for 61.5% of all accidents, with falls occurring most frequently in the home, particularly in the bathroom, bedroom, and living room, which are the main areas of activity [12]. In a South Korean study of older adults hospitalized for falls from 2015 to 2020, 57.3% of falls occurred at home, followed by streets or roads (15.4%), healthcare facilities (4.0%), congregate living facilities (3.7%), and industrial and construction sites (1.7%) [26]. In a study of older adult patients presenting to the emergency department of a level 1 trauma center in New York City, 71.3% of falls occurred indoors and 28.7% occurred outdoors [27]. For older adults, declining cognitive function and balance can reduce their ability to react to unexpected situations, including medication use that can cause dizziness or low blood pressure, and poor vision [12,24]. Installing non-slip pads and grab bars, removing moisture from toilets, and proactively educating older adults will help prevent accidents in their homes [28].

Major diseases that contribute to dysphagia are significantly more common in older adults, and dysphagia is increased due to dysfunction of the throat and esophageal motility [29]. Although the proportion of older adults presenting with airway obstruction injuries in this study was not a large, the risk of mortality for these patients was approximately 14 times higher than that for contact injuries. In particular, the most common causes of airway obstruction by foreign bodies are meat, bread, and rice cakes [30], and it is necessary to spread awareness of the risk of airway obstruction by meat and bread, which are commonly consumed in daily life, and to educate family members and caregivers of older adults with conditions that may increase the risk of airway obstruction (e.g., cerebral infarction, Parkinson’s disease, and mental illness) on how to provide first aid for choking and the need for CPR training. Furthermore, regular screening for dysphagia is recommended to prevent airway obstruction caused by foreign bodies. Preventive measures such as ensuring adequate meal times to reduce impulsive eating behaviors, eating small and frequent meals, providing a quiet eating environment, and maintaining strict oral healthcare should also be followed [31].

In this study, the ISS in older adult trauma patients was positively correlated with the risk of mortality, which is consistent with the findings of previous studies [19,32,33] that found the ISS to be the strongest predictor of mortality. A meta-analysis of predictors of mortality in older adult trauma patients also found that mortality was 50 times higher for severe injuries [17]. The ISS can effectively help healthcare providers make accurate judgments in emergency situations quickly and can be actively used for communication and treatment prioritization [34,35]. Therefore, strategies are needed to reduce mortality in older adult patients by accurately determining the extent of trauma early to provide appropriate treatment.

This study found that older adults presenting with injuries had approximately twice the odds of mortality if they received a blood transfusion compared with if they did not. Decreased physiologic function, comorbidities, and medications in older adults may be risk factors, and even small transfusions of blood products may increase adverse effects such as inflammatory marker levels, coagulopathy, immunosuppression, and the potential for infection [36]. In contrast, previous studies comparing blood loss and transfusion in critically ill older adult trauma patients with younger patients have reported that the possibility of massive transfusion should be considered in older adult patients, even if their initial assessment is unremarkable, and that liberal transfusion may result in better outcomes compared with restrictive transfusion strategies [37]. Individuals who have lost more blood due to their injury are more likely to require a transfusion. However, since this study only looked at transfusion status, it is recommended to look at how the type of blood product, the amount of blood transfused, and the timing of transfusion affect mortality in future studies.

This study had some limitations. First, because records used in the analysis were national statistics from South Korea, caution should be exercised when generalizing our results to other countries or populations. Further studies are needed to validate whether or not our findings are applicable in other countries. Second, this was a retrospective study using medical records, so the data may not have been strictly accurate, and bias may have occurred due to missing data for some variables. Third, this study did not include various risk factors for mortality, such as comorbidities [38], the health status of the individuals, medication histories [18], lengths of hospitalization [32], socio-economic class [8], and physiological variables [17]. Since data were based on patients who were transported to a medical center by a 911 ambulance, the study did not include patients with severe trauma who drove themselves or used a private ambulance. Therefore, this may have led to potential selection bias. Nevertheless, this study is significant in that it identified the most frequent in-home injury-related characteristics and mortality-related factors among older adults.

## 5. Conclusions

This study aimed to identify the clinical characteristics and mortality risk factors associated with accidental home injuries in adults aged 65 years and older to provide a basis for injury prevention strategies. The majority of older adults in this study who experienced accidental injuries at home were of an older age (75–84) and female, while the main types of injuries were falls and slips. Risk factors for mortality included older age (≥85 years), male sex, mechanism of injury, higher severity, and receiving a blood transfusion. Based on these findings, the home and community environments where older adults live should be inspected and monitored, and in-home accidental injury prevention strategies should be developed that are tailored to the characteristics of older adults’ risk factors and their injury-related characteristics.

## Figures and Tables

**Table 1:** Characteristics of elderly with unintentional injuries at home (*n* = 2153).

Characteristics	Total	Survival (*n* = 1834)	Death (*n* = 319)
	*n* (%)	*n* (%)	*n* (%)
Age			
65–74	697 (32.4)	601 (32.8)	96 (30.1)
75–84	921 (42.8)	788 (43.0)	133 (41.7)
≥85	535 (24.8)	445 (24.3)	90 (28.2)
Sex			
Female	1137 (52.8)	1001 (54.6)	136 (42.6)
Male	1015 (47.2)	832 (45.4)	183 (57.4)
Mechanism of injury			
Contact injury	317 (14.7)	299 (16.3)	18 (5.6)
Falls or slips	1465 (68.0)	1297 (70.7)	168 (52.7)
Airway obstruction	182 (8.5)	79 (4.3)	103 (32.3)
Others	189 (8.8)	159 (8.7)	30 (9.4)
Activity at injury			
Working	150 (7.0)	123 (6.7)	27 (8.5)
Daily living	1470 (68.3)	1267 (69.1)	203 (63.6)
Walking	128 (5.9)	110 (6.0)	18 (5.6)
Others	405 (18.8)	334 (18.2)	71 (22.3)
Injury severity score			
Mild (1–8)	831 (38.6)	809 (44.1)	22 (6.9)
Moderate (9–15)	547 (25.4)	511 (27.9)	36 (11.3)
Severe (16–24)	104 (4.8)	75 (4.1)	29 (9.1)
Very severe (≥25)	288 (13.4)	94 (5.1)	194 (60.8)
Unknown	383 (17.8)	345 (18.8)	38 (11.9)
Surgery			
No	1782 (82.8)	1515 (82.6)	267 (83.7)
Yes	371 (17.2)	319 (17.4)	52 (16.3)
Transfusion			
No	1919 (89.1)	1663 (90.7)	256 (80.3)
Yes	145 (6.7)	101 (5.5)	44 (13.8)
Unknown	89 (4.1)	70 (3.8)	19 (6.0)

**Table 2:** Single-variable and multiple logistic regression: mortality (*n* = 2153).

Characteristics	Single-Variable Logistic Regression		Multiple Logistic Regression	
	Odds Ratio [95% CI]	*p*	Odds Ratio [95% CI]	*p*
Age				
65–74	1		1	
75–84	1.06 [0.80–1.40]	0.703	1.26 [0.86–1.83]	0.231
≥85	1.27 [0.93–1.73]	0.139	2.25 [1.47–3.45]	<0.001
Sex				
Female	1		1	
Male	1.62 [1.27–2.06]	<0.001	1.60 [1.15–2.20]	0.005
Mechanism of injury				
Contact injury	1		1	
Falls or slips	2.15 [1.30–3.56]	0.003	6.76 [3.39–13.47]	<0.001
Airway obstruction	21.66 [12.39–37.87]	<0.001	13.96 [6.35–30.71]	<0.001
Others	3.13 [1.69–5.80]	<0.001	4.18 [2.04–8.55]	<0.001
Activity at injury				
Working	1		1	
Daily living	0.73 [0.47–1.14]	0.730	0.80 [0.44–1.46]	0.459
Walking	0.75 [0.39–1.43]	0.745	0.98 [0.43–2.22]	0.960
Others	0.97 [0.59–1.58]	0.968	1.64 [0.87–3.09]	0.127
Injury severity score				
Mild (1–8)	1			
Moderate (9–15)	2.59 [1.51–4.45]	0.001	2.56 [1.45–4.54]	0.001
Severe (16–24)	14.22 [7.78–25.97]	<0.001	12.24 [6.48–23.12]	<0.001
Very severe (≥25)	75.89 [46.50–123.87]	<0.001	67.95 [38.86–118.81]	<0.001
Unknown	4.05 [2.36–6.95]	<0.001	14.45 [7.38–28.27]	<0.001
Surgery				
No	1			
Yes	0.93 [0.67–1.28]	0.633	0.71 [0.44–1.15]	0.164
Transfusion				
No	1			
Yes	2.83 [1.94–4.13]	<0.001	2.14 [1.24–3.67]	0.006
Unknown	1.76 [1.04–2.98]	0.034	1.80 [0.89–3.61]	0.102

## Data Availability

The data that support the findings of this study are available from the Korea Disease Control and Prevention Agency (KDCA), but restrictions apply to the availability of these data, which were used under a license for the current study, and therefore they are not publicly available.
